# Individual i*n vitro* effects of ochratoxin A, deoxynivalenol and zearalenone on oxidative stress and acetylcholinesterase in lymphocytes of broiler chickens

**DOI:** 10.1186/2193-1801-3-506

**Published:** 2014-09-08

**Authors:** Claudia Lautert, Laerte Ferreiro, Patrícia Wolkmer, Francine C Paim, Cássia B da Silva, Jeandre AS Jaques, Sônia TA Lopes, Janio M Santurio

**Affiliations:** Setor de Micologia, Faculdade de Veterinária (FAVET), Universidade Federal do Rio Grande do Sul (UFRGS), Av. Bento Gonçalves, 9090, 91540-000 Porto Alegre, RS Brasil; Curso de Medicina Veterinária, Universidade de Cruz Alta (UNICRUZ), Campus Universitário Dr. Ulysses Guimarães - Rodovia Municipal Jacob Della Méa, Km 5.6, 98020-290 Cruz Alta, RS Brasil; Laboratório de Análises Clínicas Veterinário (LACVET), Universidade Federal de Santa Maria (UFSM), Avenida Roraima, 1000, Cidade Universitária, Bairro Camobi, 97105-900 Santa Maria, RS Brasil; Universidade Federal de Mato Grosso do Sul (UFMS), Cidade Universitária, 79070-900 Campo Grande, MS Brasil; Departamento de Microbiologia e Parasitologia, Laboratório de Pesquisas Micológicas (LAPEMI), Universidade Federal de Santa Maria (UFSM), Avenida Roraima, 1000, Cidade Universitária, Bairro Camobi, 97105-900 Santa Maria, RS Brasil

**Keywords:** Cytotoxicity, Enzymatic activity, Oxidative burst, Leucocytes, Lipid peroxidation, Mycotoxins

## Abstract

The contamination of consumer food and animal feed with toxigenic fungi has resulted in economic losses worldwide in animal industries. Mycotoxins are highly biologically reactive secondary metabolites and can inhibit protein synthesis and cell multiplication. Considering the cytotoxicity of mycotoxins, this experiment was performed to determine the *in vitro* influence of ochratoxin A, deoxynivalenol and zearalenone on lipid peroxidation in lymphocytes of broiler chickens at different concentrations. This study has also evaluated whether the presence of these mycotoxins changes the acetylcholinesterase activity in lymphocytes, which is involved in the regulation of immune and inflammatory responses. Blood lymphocytes of broiler chickens were isolated through density gradient centrifugation and incubated with the respective mycotoxins at concentrations of 0.001, 0.01, 0.1 and 1 μg/mL. Lipid peroxidation, which was evaluated through the amount of malondialdehyde measured in a thiobarbituric acid-reactive species test, and the enzymatic activity were analyzed at 24, 48 and 72 h. Results of the lipid peroxidation evaluation showed an increasing cytotoxicity relation: ochratoxin A > deoxynivalenol > zearalenone. Conversely, cytotoxicity was valued as zearalenone > deoxynivalenol > ochratoxin A in relation to the acetylcholinesterase enzymatic activity. At a concentration of 1 μg/mL, ochratoxin A and deoxynivalenol induced the highest cellular oxidative stress levels and the highest enzymatic activity at the majority of time points. However, the same mycotoxins, except at 1 μg/mL concentration, induced a reduction of lymphocytic lipid peroxidation 72 h after incubation, suggesting the action of a compensatory mechanism in these cells.

## Introduction

Mycotoxins are fungal secondary metabolites present in 25% of the grains produced worldwide (Santurio 2000). The exposure to these secondary metabolites occurs through the ingestion of contaminated products, leading to a number of serious health problems, including immunosuppression and carcinogenesis (Keller et al. 2005).

Reactive oxygen species (ROS) are associated to several molecular changes on cellular components, resulting in cellular morphology and viability alterations. High levels of ROS may promote cell oxidative damage, such as DNA injury, protein oxidation and lipid peroxidation (Zhang et al. 2009).

Acetylcholinesterase (AChE) is an enzyme present in the lymphocytic membrane and cytoplasm responsible for regulating acetylcholine (ACh), which modulates the activation and differentiation of lymphocytes via extraneuronal cholinergic system (Wessler and Kirkpatrick 2001). The ACh released by lymphocytes may have immunomodulatory action through muscarinic or nicotinic ACh receptors (Kawashima and Fujii 2000). Since the interaction between ACh and its receptor depends on the catalytic efficiency of AChE, the activity of this enzyme can be used as a rate of the cholinergic function because changes in its activity may indicate alterations in the availability of ACh receptors (Bennedito 1997).

Mycotoxins can induce genotoxic and cytotoxic effects and may be associated to cellular oxidative stress; consequently, the lipid peroxidation induced by mycotoxins could alter the AChE activity. In order to determine and compare the mycotoxin cytotoxicity, lymphocytes of broiler chickens were incubated *in vitro* with different concentrations of ochratoxin A, deoxynivalenol and zearalenone mycotoxins. Lipid peroxidation levels were analyzed using thiobarbituric acid-reactive species (TBARS) test, and AChE was quantified.

## Materials and methods

### Reagents

All reagents used in the experiments were of analytical grade and of the highest purity: acetylthiocholine iodide, 5,5’-dithio-bis-2-nitrobenzoic acid, tris-(hydroxymethyl)-aminomethane GR, Coomassie brilliant blue G, RPMI 1640 cell culture medium, fetal bovine serum (FBS), penicillin and streptomycin (Sigma Chemical Co., St. Louis, MO, USA).

### Mycotoxins

The mycotoxins used were ochratoxin A (OTA), deoxynivalenol (DON) and zearalenone (ZON) (Sigma Chemical Co., St. Louis, MO, USA). They were solubilized in ethanol (5%) at concentrations of 0.001, 0.01, 0.1 and 1 μg/mL and were added to lymphocyte cultures for respective assessment.

### Cells and culture

Lymphocytes were isolated from broiler chicken blood in accordance with the guidelines for the ethical conduct in the care and use of animals of Universidade Federal de Santa Maria (UFSM), Brazil. The blood pool was collected from jugular vein of 45-day-old Coob 500 lineage poultry using Falcon conical tubes with 10% EDTA, and the cell isolation technique was performed through density gradient centrifugation as described by Boyum (1968). After the counting of cells in a Neubauer chamber, stained with trypan blue 0.1%, concentrations of 0.7 × 10^5^ lymphocytes/mL were cultured in RMPI 1640 medium, supplemented with 10% FBS and 2.5 IU/mL penicillin/streptomycin and maintained at 37°C in 5% CO_2_. The suspension of cells was placed in 96-well plates and maintained in exponential growth (80% confluence). Each mycotoxin (20 μL) was added to the cell cultures at different concentrations (0.001, 0.01, 0.1 and 1 μg/mL), and the analysis were carried out 24, 48 and 72 h post incubation.

### Sample preparation and protein determination

The number of cells was measured by counting in a Neubauer chamber. Lymphocytes were washed in phosphate buffer (3X), centrifuged for 10 min (1.400 rpm), and then the supernatant was discarded. The pellet was resuspended in Hanks' Balanced Salt Solution (HBSS) to a final protein concentration of 0.1 to 0.2 mg/mL. Protein concentration was determined by the Coomassie blue method (Bradford 1976), using bovine serum albumin as a standard.

### AChE activity in lymphocytes

The AChE activity was measured by adapting the technique described by Ellman et al. (1961) and modified by Fitzgerald and Costa (1993) to evaluate it in lymphocytes. Briefly, 0.2 mL of each sample was added to a solution containing 1.0 mM acetylthiocholine (AcSCh), 0.1 mM 5,5′-dithiobis (2-nitrobenzoic acid) (DTNB) and 100 mM phosphate buffer (pH 8.0). Absorbance was read on a spectrophotometer at 412 nm. HBSS was used as a negative control. AChE values were calculated from the AChE activity and the protein content, and results were expressed as nmol of h/mg of protein.

### Lipid peroxidation

Cell lipid peroxidation was measured using TBARS levels, as described by Jentzsch et al. (1996). Results were obtained by spectrophotometry at 535 nm and expressed as nmol of MDA/mg of protein.

### Statistical analysis

Differences between the treated groups were determined by one-way ANOVA followed by the Newman-Keuls post test, considering P < 0.05 as a level significance. The experiment was replicated twice, and the samples were measured in triplicate. Results were expressed as mean ± standard error of the mean.

## Results

### Ochratoxin A

**Figure 1:**
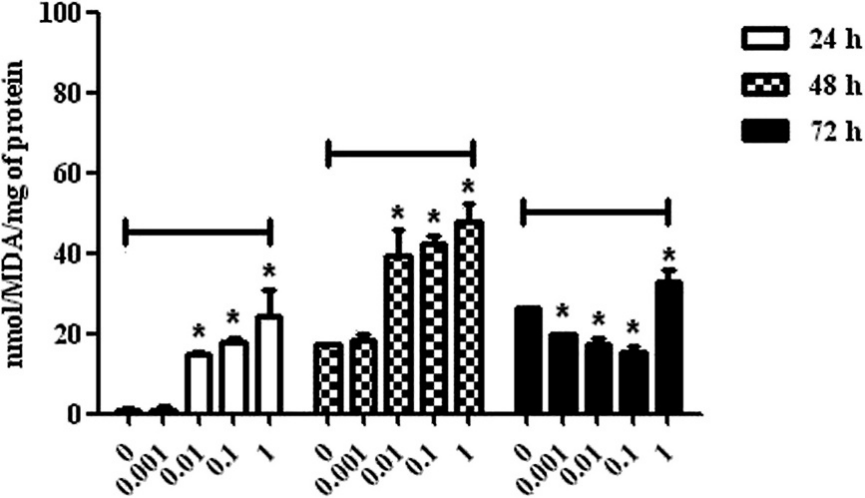
Malondialdehyde levels in broiler chickens lymphocytes exposed to ochratoxin A at concentrations of 0, 0.001, 0.01, 0.1 and 1 μg/mL at 24, 48 and 72 h. P < 0.05 (*).

**Figure 2:**
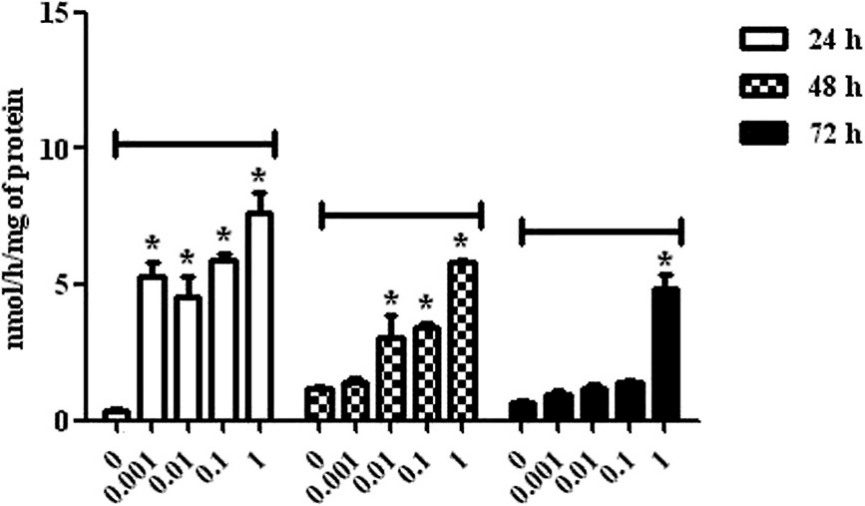
Acetylcholinesterase activity in broiler chickens lymphocytes exposed to ochratoxin A at concentrations of 0, 0.001, 0.01, 0.1 and 1 μg/mL at 24, 48 and 72 h. P < 0.05 (*).

After the incubation with OTA mycotoxin, lymphocytic cells showed an increase in malondialdehyde (MDA) production when compared to the control group, which resulted in higher lymphocyte cytotoxicity in a dose-responsive manner in 0.01 μg/mL (15.00 ± 0.57 and 39.26 ± 6.72), 0.1 μg/mL (17.73 ± 1.04 and 42.09 ± 2.41) and 1 μg/mL (24.42 ± 6.21 and 47.86 ± 4.71) concentrations analyzed in the first 48 h. However, the OTA concentration of 0.001 μg/mL did not induce significant MDA levels in lymphocytic cells until 48 h. At 72 h, there was a decrease of these levels, representing lower cell oxidative stress, with the exception of the cells incubated with the concentration of 1 μg/mL (32.97 ± 3.03) (P < 0.05). The 0.1 μg/mL concentration induced the lowest MDA levels (15.43 ± 1.31) in the cells 72 h post-incubation (P < 0.05) (Figure 1).The AChE analysis revealed an increase in the enzymatic activity in lymphocytes incubated with OTA 24 h post-incubation fort all concentrations when compared to the control group (P < 0.05). After 48 h, only the lymphocytes incubated with the concentration of 0.001 μg/mL did not demonstrate significant levels of AChE. After the full period of analysis, this increase remained only in the cells incubated with the concentration of 1 μg/mL (4.82 ± 0.57) (P < 0.05) (Figure 2).

### Deoxynivalenol

**Figure 3:**
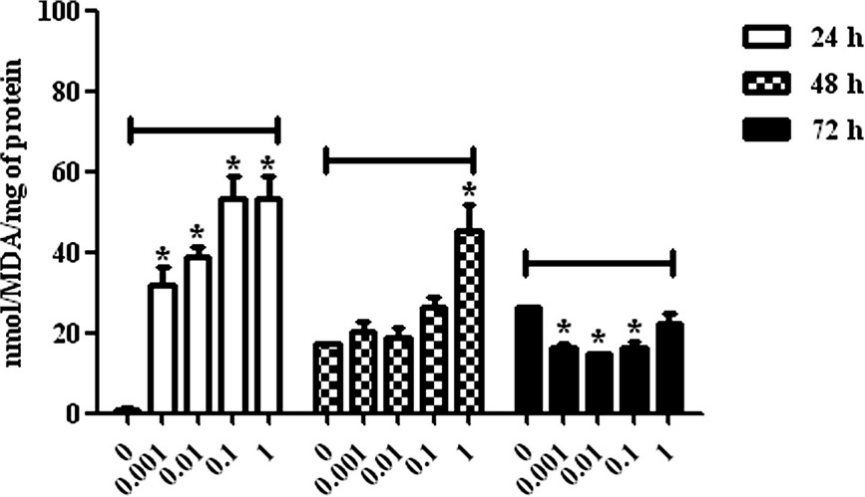
Malondialdehyde levels in broiler chickens lymphocytes exposed to deoxynivalenol at concentrations of 0, 0.001, 0.01, 0.1 and 1 μg/mL at 24, 48 and 72 h. P < 0.05 (*).

**Figure 4:**
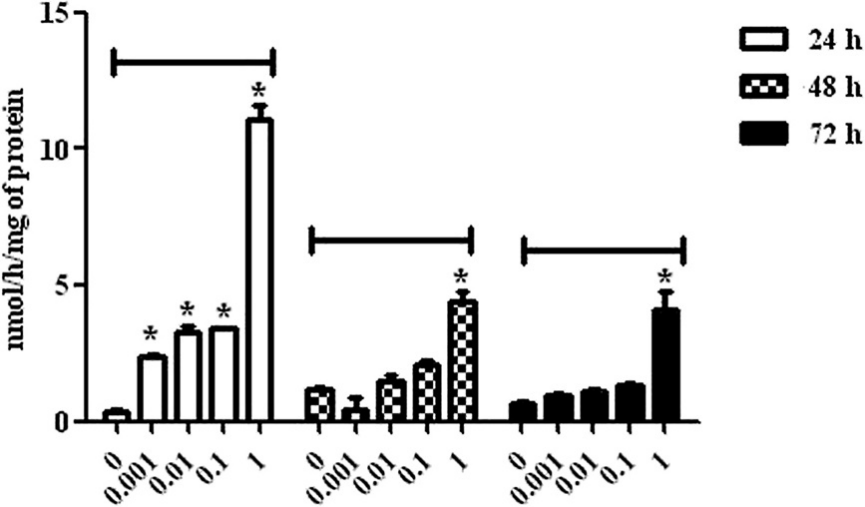
Acetylcholinesterase activity in broiler chickens lymphocytes exposed to deoxynivalenol at concentrations of 0, 0.001, 0.01, 0.1 and 1 μg/mL at 24, 48 and 72 h. P < 0.05 (*).

Regarding MDA levels in lymphocytes incubated with DON, there was an increase in the lipid peroxidation level observed in all concentrations after 24 h of incubation in comparison with the control group (P < 0.05). After 48 h, cell oxidative damage was observed only at the highest concentration, 1 μg/mL (45.35 ± 6.31), whereas at 72 h, there was a decrease in the oxidative stress level in the cells incubated with the concentrations 0.001 μg/mL (16.50 ± 0.86), 0.01 μg/mL (14.65 ± 0.27) and 0.1 μg/mL (16.26 ± 1.74) (P < 0.05) (Figure 3).The AChE evaluation at 24 h demonstrated an increase of the enzymatic activity in the cells incubated with DON at all concentrations when compared to the control group (P < 0.05), and those incubated with 1 μg/mL mycotoxin concentration presented the highest AChE activity (11.08 ± 0.50) (P < 0.05). After 48 and 72 h, only the lymphocytes incubated with 1 μg/mL mycotoxin concentration presented significant levels of AChE activity, represented respectively by 4.37 ± 0.43 and 4.07 ± 0.69 (P < 0.05) (Figure 4).

### Zearalenone

**Figure 5:**
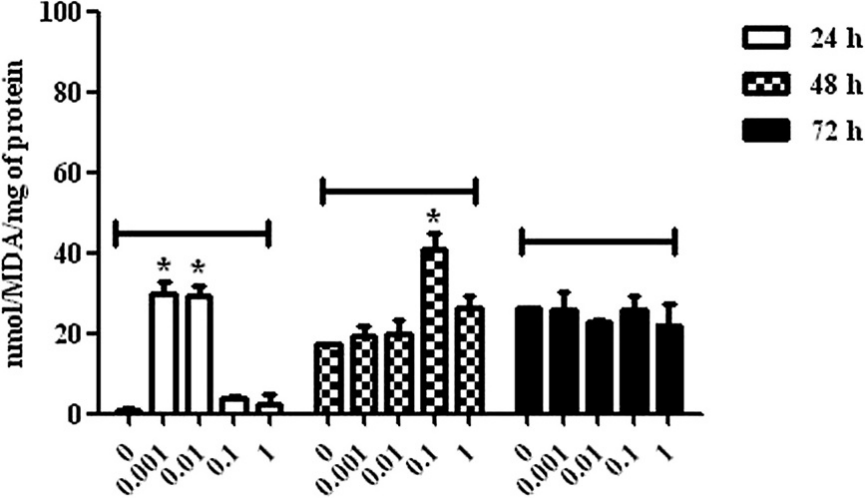
Malondialdehyde levels in broiler chickens lymphocytes exposed to zearalenone at concentrations of 0, 0.001, 0.01, 0.1 and 1 μg/mL at 24, 48 and 72 h. P < 0.05 (*).

**Figure 6:**
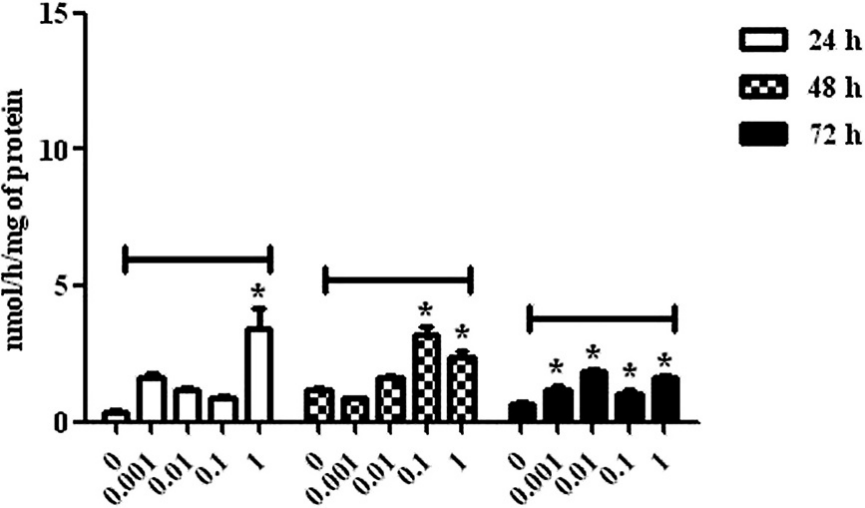
Acetylcholinesterase activity in broiler chickens lymphocytes exposed to zearalenone at concentrations of 0, 0.001, 0.01, 0.1 and 1 μg/mL at 24, 48 and 72 h. P < 0.05 (*).

The lowest concentrations of mycotoxin (0.001 and 0.01 μg/mL) caused an increase in MDA levels (30.00 ± 2.88 and 29.18 ± 2.76, respectively) in lymphocytic cells at 24 h (P < 0.05), whereas the 0.1 μg/mL concentration caused an increase at 48 h (41.01 ± 3.92) (P < 0.05), when compared to the control group. A significant increase in MDA levels was not observed at 72 h, indicating that the oxidative stress was a resulted of the natural process of cellular oxidation, as observed in the control group (Figure 5).The AChE analysis demonstrated an increase of the enzymatic activity in cells incubated with ZON at 1 μg/mL concentration at 24 h (3.41 ± 0.77) (P < 0.05) and, at 0.1 (3.18 ± 0.34) and 1 (2.38 ± 0.23) μg/mL concentrations at 48 h (P < 0.05). At 72 h, all concentrations of ZON caused an increase of the AChE activity in comparison to the control group (P < 0.05) (Figure 6).

## Discussion

Oxidative stress is a term used to define the outcome of an imbalance between the prooxidant (ROS) and antioxidant molecules, which results in wide range cell damages. In order to assess oxidative stress, methods that quantify peroxidation products and antioxidant agents are available (Hassen et al. 2007). MDA was chosen as a parameter of oxidative stress and cellular injury in this study because it is an end-product of the lipid peroxidation, which is one of the cellular pathways involved in oxidative damage and it is associated to cytotoxicity induced by mycotoxins (Abid-Essefi et al. 2004).

Despite the effect of the cholinergic system on lymphocytes remains unclear, the demonstration of the existence of cholinergic markers in lymphocytes may help assess the importance of the cholinergic system as a possible regulator of the immune system (Tayebati et al. 2002). In addition to performing lipid peroxidation analysis, this study also evaluated the *in vitro* AChE activity in lymphocytes of broiler chickens after incubation with different concentrations of OTA, DON and ZON mycotoxins.

The evaluation of AChE activity revealed DON to be more cytotoxic to the lymphocytes of broiler chickens than OTA and less cytotoxic than ZON. The lymphocytic cells added to 1 μg/mL DON concentration presented the highest levels of AChE activity, and the same result was also observed in the AChE activity analysis post-incubation with OTA.

According to Paterson and Lima (2010), the probable primary biochemical lesions and early cellular events leading to toxic cell injury or cellular deregulation associated with OTA exposure would be in the following sequence: disruption of phenylalanine metabolism, reduction of PEPCK (phosphoenolpyruvate carboxykinase), reduction of gluconeogenesis and cell death. The cell death may occur by two alternative routes: through metabolic activation, inhibition of protein synthesis (DNA) and apoptosis, or through the alteration of membrane permeability and disruption of calcium homeostasis, leading to cell deregulation. These cellular events could explain the process that occurred mainly in the first 48 h of this study, but they do not justify the decreased oxidative stress levels at 72 h.

The decrease of OTA lipid peroxidation levels within 72 h was similar to that observed in cells incubated with DON, suggesting a compensatory mechanism that is indicative of an autoregulatory process in these immunological cells. According to the study of Kamimura et al. (2003), ACh stimulates nitric oxide synthesis in CCRF-CEM cells (Human T cell lymphoblast-such as cell line), which could be related to increased cytotoxicity. The relation between ACh and AChE is inversely proportional, which means that the higher the activity of AChE, the lower the amount of acetylcholine available in the cell. Thus, unlike the study by Kamimura et al. (2003), our study found no relation between the activity of AChE with decreased lipid peroxidation 72 h post-incubation of lymphocytes of broiler chickens with OTA and DON, since the cells did not show significant levels of the enzyme.

Klarić et al. (2006) observed that OTA at 5 μg/mL induced high levels of stress oxidative in porcine kidney PK15 cells at 24 h, with further significant increase after 48 h exposure, whereas the other concentrations analyzed, 0.5 and 0.05 μg/mL, showed no significant results. Our study demonstrated similar OTA sensitivity to a primary culture of lymphocytes of broiler chickens, but at lower concentrations.

DON has been reported to induce lipid peroxidation using Caco-2 cells (Kouadio et al., 2005) and elevate TBARS levels in a dose-responsive manner in human hepatoma HepG2 cells (Zhang et al., 2009). This production of radical species associated to oxidative stress could cause DNA injury. The same dose-responsive manner showed by the study of Zhang et al. (2009) was demonstrated in our study only in the first 24 h of analysis.

According to Bondy and Pestka (2000), trichotecenes affect the humoral immunity and can act as either stimulators or suppressors of the immune system based on some variables such as the dose, frequency and the time of exposure. In this study, DON demonstrated lower cytotoxicity through lipid peroxidation and higher enzymatic inhibition than did OTA. Compared to zearalenone, DON caused higher lipid peroxidation and lower enzymatic inhibition.

Several studies have demonstrated the cytotoxic effects of ZON, such as the inhibition of cellular proliferation and synthesis of macromolecules in different cell lines (Severino et al. 2008, Abid-Essefi et al. 2004), induction of lipid peroxidation, and cell death (Abid-Essefi et al. 2004). Moreover, genotoxic effects such as apoptosis induction, production of DNA fragmentation (Abid-Essefi et al. 2003, Kim et al. 2003) as well as micronuclei (Ouanes et al. 2003) and chromosome aberrations (IARC International Agency for Research on Cancer 1993, Ouanes et al. 2003) have also been presented.

In poultry, the hepatic biotransformation of the mycotoxin ZON results in the main product β-zearalenol (β-ZOL) (Gajęcki et al. 2010), which is considered an inactivation reaction (Fitzpatrick et al. 1989; Leffers et al. 2001), since β-ZOL is generally three times less estrogenic than α-ZOL (Wyatt 1991). In our *in vitro* study, the ZON analysis in lymphocytes of broiler chickens demonstrated lower cytotoxicity in comparison to the other mycotoxins assessed, since the cells used in our study were not specifically target for ZON. This result was in agreement with the data previously obtained in CHO-K1 cells (Cetin and Bullerman 2005, Ferrer et al. 2009) and in other cell lines (Hassen et al. 2007, Ayed-Boussema et al. 2008, Bouaziz et al. 2008). Nevertheless, Abid-Essefi et al. (2004) showed that ZON induced oxidative damage by enhancing lipid peroxidation on nonspecific target cell line Vero and Caco-2 cells, since the mycotoxin increased MDA formation in a concentration-dependent manner.

## Conclusions

*In vitro* cell culture assays have contributed to mycotoxin research through supplementary information on biochemical mechanisms of cytotoxicity of these metabolites. In the assessment of MDA levels and the consequent lipid peroxidation in lymphocytes of broiler chickens exposed to different concentrations of mycotoxins *in vitro*, cytotoxicity was presented in the following order: OTA > DON > ZON. In relation to the enzymatic activity of AChE, the cytotoxicity assessment was ZON > DON > OTA. The inversely proportional relation of the cytotoxicity assessment of mycotoxins between lipid peroxidation and AChE activity suggests that the higher the enzymatic activity, the lower the cellular oxidative stress. Nevertheless, this effect did not occur at 1 μg/mL concentration because OTA and DON mycotoxins showed to induce the highest levels of cellular oxidative stress at most of the time points as well as the highest levels of AChE activity.

Regarding OTA and DON mycotoxins, it is important to emphasize that in the final period of assessment, 72 h, there was a decrease in the MDA levels of the lymphocytes of broiler chickens. However, this effect was not observed in cells incubated with the concentration 1 μg/mL, which resulted in less cellular oxidative stress in comparison with the initial periods of 24 and 48 h, suggesting the action of a compensatory mechanism in these cells.

## References

[CR1] Abid-Essefi S, Baudrimont I, Hassen W, Ouanes Z, Mobio TA, Anane R, Creppy EE, Bacha H (2003). DNA fragmentation, apoptosis and cell cycle arrest induced by zearalenone in cultured DOK, Vero and Caco-2 cells: prevention by Vitamin E. Toxicology.

[CR2] Abid-Essefi S, Ouanes Z, Hassen W, Baudrimont I, Creppy E, Bacha H (2004). Cytotoxicity, inhibition of DNA and protein syntheses and oxidative damage in cultured cells exposed to zearalenone. Toxicol in Vitro.

[CR3] Ayed-Boussema I, Bouaziz C, Rjiba K, Valenti K, Laporte F, Bacha H, Hassen W (2008). The mycotoxin zearalenone induces apoptosis in human hepatocytes (HepG2) via p53-dependent mitochondrial signalling pathway. Toxicol in Vitro.

[CR4] Bennedito G, O’Neill JO, Doukas PH (1997). Drogas que afetam o Sistema Nervoso Parassimpático e Gânglios Autônomos. Farmacologia Clínica.

[CR5] Bondy GS, Pestka JJ (2000). Immunomodulation by fungal toxins. J Toxicol Env Heal B.

[CR6] Bouaziz C, Sharaf el dein O, El Golli E, Abid-Essefi A, Brenner C, Lemaire C, Bacha H (2008). Different apoptotic pathways induced by zearalenone, T-2 toxin and ochratoxin A in human hepatoma cells. Toxicology.

[CR7] Boyum A (1968). Isolation of mononuclear cells and granulocytes from human blood. Isolation of mononuclear cells by one centrifugation, and of granulocytes by combining centrifugation and sedimentation at 1 g. Scand J Clin Lab Invest.

[CR8] Bradford MM (1976). A rapid and sensitive method for the quantitation of microgram quantities of protein utilizing the principle of protein-dye binding. Anal Biochem.

[CR9] Cetin Y, Bullerman LB (2005). Cytotoxicity of *Fusarium* mycotoxins to mammalian cell cultures as determined by the MTT bioassay. Food Chem Toxicol.

[CR10] Ellman GL, Courtney KD, Andres V, Feather-Stone RM (1961). A new and rapid colorimetric determination of acetylcholinesterase activity. Biochem Pharmacol.

[CR11] Ferrer E, Juan-García A, Font G, Ruiz MJ (2009). Reactive oxygen species induced by beauvericin, patulin and zearalenone in CHO-K1 cells. Toxicol in Vitro.

[CR12] Fitzgerald BB, Costa LG (1993). Modulation of muscarinic receptors and acetylcholinesterase activity in lymphocytes and in brain areas following repeated organophosphate exposure in rats. Fund Appl Toxicol.

[CR13] Fitzpatrick DW, Picken CA, Murphy LC, Buhr MM (1989). Measurement of the relative binding affinity of zearalenone, α-zearalenol and β-zearalenol for uterine and oviduct estrogen receptors in swine, rats and chickens: an indicator of estrogenic potencies. Comp Biochem Phys C.

[CR14] Gajęcki M, Gajęcka M, Jakimiuk E, Zielonka Ł, Obremski K, Raí M, Varma A (2010). Zearalenone Biotransformation. Mycotoxins in Food, Feed and Bioweapons.

[CR15] Hassen W, Ayed-Boussema I, Oscoz AA, Lopez AC, Bacha H (2007). The role of oxidative stress in zearalenone-mediated toxicity in Hep G2 cells: Oxidative DNA damage, gluthatione depletion and stress proteins induction. Toxicology.

[CR16] (1993). Some naturally occurring substances: food items and constituents, heterocyclic aromatic amines and mycotoxins. IARC Monographs on the Evaluations on the Carcinogenic Risk of Chemicals to Humans.

[CR17] Jentzsch AM, Bachmann H, Furst P, Biesalski HK (1996). Improved analysis of malondialdehyde in human body fluids. Free Radical Bio Med.

[CR18] Kamimura Y, Fujii T, Kojima H, Nagano T, Kawashima K (2003). Nitric oxide (NO) synthase mRNA expression and NO production via muscarinic acetylcholine receptor-mediated pathways in the CEM, human leukemic T-cell line. Life Sci.

[CR19] Kawashima K, Fujii T (2000). Extraneuronal cholinergic system in lymphocytes. Pharmacol Therapeut.

[CR20] Keller NP, Turner G, Bennett JW (2005). Fungal secondary metabolism – from biochemistry to genomics. Nat Rev Microbiol.

[CR21] Kim IH, Son HY, Cho SW, Ha CS, Kang BH (2003). Zearalenone induces male germ cell apoptosis in rats. Toxicol Lett.

[CR22] Klarić MŠ, Pepeljnjak S, Domijan A, Petrik J (2006). Lipid peroxidation and glutathione levels in porcine kidney PK15 cells after individual and combined treatment with Fumonisin B1, Beauvericin and Ochratoxin A. Basic Clin Pharmacol.

[CR23] Kouadio JH, Mobio TA, Baudrimont I, Moukha S, Dano SD, Creppy EE (2005). Comparative study of cytotoxicity and oxidative stress induced by deoxynivalenol, zearalenone or fumonisin B_1_ in human intestinal cell line Caco-2. Toxicology.

[CR24] Leffers H, N_sby M, Vendelbo B, Skakkeb_k NE, Jørgensen M (2001). Oestrogenic potencies of zeranol, oestradiol, diethylstilboestrol, bisphenol-A and genistein: implications for exposure assessment of potential endocrine disrupters. Hum Reprod.

[CR25] Ouanes Z, Abid S, Ayed I, Anane R, Mobio T, Creppy E, Bacha H (2003). Induction of micronuclei by zearalenone in Vero monkey kidney cells and in bone marrow cells of mice: protective effect of vitamin E. Mutat Res.

[CR26] Paterson RRM, Lima N (2010). Toxicology of mycotoxins. Mol Clin Environ Toxicol.

[CR27] Santurio JM (2000). Micotoxinas e micotoxicoses na avicultura. Rev Bras Cienc Avic.

[CR28] Severino L, Russo R, Luongo D, De Luna R, Ciarcia R, Rossi M (2008). Immune effects of four *Fusarium*-toxins (FB1, ZEA, NIV, DON) on the proliferation of Jurkat cells and porcine lymphocytes: *in vitro* study. Vet Res Commun.

[CR29] Tayebati SK, Amenta F, El-Assouad D, Zaccheo D (2002). Muscarinic cholinergic receptor subtypes in the hippocampus of aged rats. Mech Ageing Dev.

[CR30] Wessler I, Kirkpatrick CJ, Zaagsma J, Meurs H, Roffel AF (2001). Role of non-neuronal and neuronal acetylcholine in the airways. Muscarinic receptors in airways diseases.

[CR31] Wyatt RD, Smith JE, Henderson RS (1991). Zearalenone toxicosis (F-2 toxicosis) in poultry. Mycotoxins and animal foods.

[CR32] Zhang X, Jiang L, Geng C, Cao J, Zhong L (2009). The role of oxidative stress in deoxynivalenol-induced DNA damage in HepG2 cells. Toxicon.

